# Hyperammonemia: What Urea-lly Need to Know: Case Report of Severe Noncirrhotic Hyperammonemic Encephalopathy and Review of the Literature

**DOI:** 10.1155/2016/8512721

**Published:** 2016-09-21

**Authors:** Ruby Upadhyay, Thomas P. Bleck, Katharina M. Busl

**Affiliations:** ^1^Department of Neurological Sciences, Rush University Medical Center, 1725 West Harrison Street, POB Suite 1121, Chicago, IL 60612, USA; ^2^Rush Medical College, Rush University Medical Center, 600 S. Paulina Street, Chicago, IL 60612, USA; ^3^Department of Neurology, Division of Neurocritical Care, University of Florida, McKnight Brain Institute, Room L3-100, 1149 Newell Drive, Gainesville, FL 32611, USA

## Abstract

*Purpose*. A 66-year-old man who presented with coma was found to have isolated severe hyperammonemia and diagnosed with a late-onset urea-cycle disorder. He was treated successfully and had full recovery.* Methods*. We report a novel case of noncirrhotic hyperammonemia and review the literature on this topic. Selected literature for review included English-language articles concerning hyperammonemia using the search terms “hyperammonemic encephalopathy”, “non-cirrhotic encephalopathy”, “hepatic encephalopathy”, “urea-cycle disorders”, “ornithine transcarbamylase (OTC) deficiency”, and “fulminant hepatic failure”.* Results*. A unique case of isolated hyperammonemia diagnosed as late-onset OTC deficiency is presented. Existing evidence about hyperammonemia is organized to address pathophysiology, clinical presentation, diagnosis, and treatment. The case report is discussed in context of the reviewed literature.* Conclusion*. Late-onset OTC deficiency presenting with severe hyperammonemic encephalopathy and extensive imaging correlate can be fully reversible if recognized promptly and treated aggressively.

## 1. Background

Ammonia is a highly potent neurotoxin well known for its implication in hepatic encephalopathy [1]. Any case of acute altered mental status should prompt a consideration of hyperammonemia as a potential cause. When hyperammonemia is detected, the etiology of its accumulation must be determined in order to guide treatment. While hyperammonemia in adults is related to severe, often cirrhotic, liver disease in 90% of cases [2], increased ammonia production or decreased ammonia elimination is one of the alternative etiologies of hyperammonemia [1]. Awareness of hyperammonemia in absence of severe hepatic disease may lead to lifesaving prompt diagnosis and treatment.

We present a case of acute severe noncirrhotic hyperammonemia and review its pathophysiology, diagnostic, and treatment considerations.

## 2. Case Presentation

A 66-year-old male business manager with past medical history of hypertension, diabetes mellitus, and intermittent sinusitis was transferred to our tertiary care center for progressive encephalopathy and concern for nonconvulsive status epilepticus.

Three weeks prior to admission, he was prescribed a 10-day course of levofloxacin for presumed sinusitis. As cough and postnasal drip did not improve, he was treated with two courses of methylprednisolone in addition to levofloxacin and subsequently clarithromycin. Two days prior to presentation, he developed headaches and sinus pressure and complained about “feeling off,” epigastric pain, and dry heaving. The night prior to presentation, he awoke frequently throughout the night with progressive confusion. The patient did not have fever, chills, or sweats. His family reported a history of anxiety, panic attacks, claustrophobia, and anger outbursts, with the last notable outburst having occurred about 6 months earlier. Of note, coworkers found a collection of various over-the-counter vitamins and nutritional supplements at the patient's desk.

Given increasing confusion and agitation, the patient was taken to another hospital where he became progressively more obtunded, requiring intubation for airway protection. He was empirically started on vancomycin, ceftriaxone, and acyclovir for concern of infectious meningoencephalitis. CSF analysis revealed elevated total protein at 53 mg/dL, glucose of 160 mg/dL (serum glucose: 240 mg/dL), no pleocytosis (1 WBC/mm^3^), negative CSF VDRL, negative West nile virus IgM and IgG, negative cryptococcal antigen, and negative fungal and bacterial cultures. Due to development of teeth grinding and concern for subclinical seizure activity, a routine electroencephalogram (EEG) was performed that revealed no seizure activity, however findings consistent with global cerebral dysfunction. He was started on phenytoin and subsequently on levetiracetam. An MRI of the brain showed chronic mild periventricular white matter hyperintensities but no acute findings. He was transferred to our tertiary care center on hospital day 3 for concern of nonconvulsive status epilepticus.

On examination, he was intubated and mechanically ventilated, with no abnormal general physical findings. On neurological examination, he was comatose, with intact brainstem reflexes and extensor posturing in all extremities to central stimulation. Initial laboratory work was notable for normal liver function panel, serum ammonia 120 *μ*g/dL (reference range 65–107 *μ*g/dL), and negative hepatitis panel, as well as respiratory alkalosis. Repeat CSF analysis showed 3 WBC/mm^3^ (76% neutrophils), 1 RBC/mm^3^, lactic acid 2.6 meq/L, total protein 28.5 mg/dL, and negative herpes simplex virus PCR as well as negative enterovirus panel, and acyclovir was discontinued. Treatment with lactulose was begun, but serum ammonia rapidly rose to 494 *μ*g/dL within a few hours of arrival to our hospital. Rifaximin and L-carnitine were added, and emergent hemodialysis was initiated.

**Figure 1:**
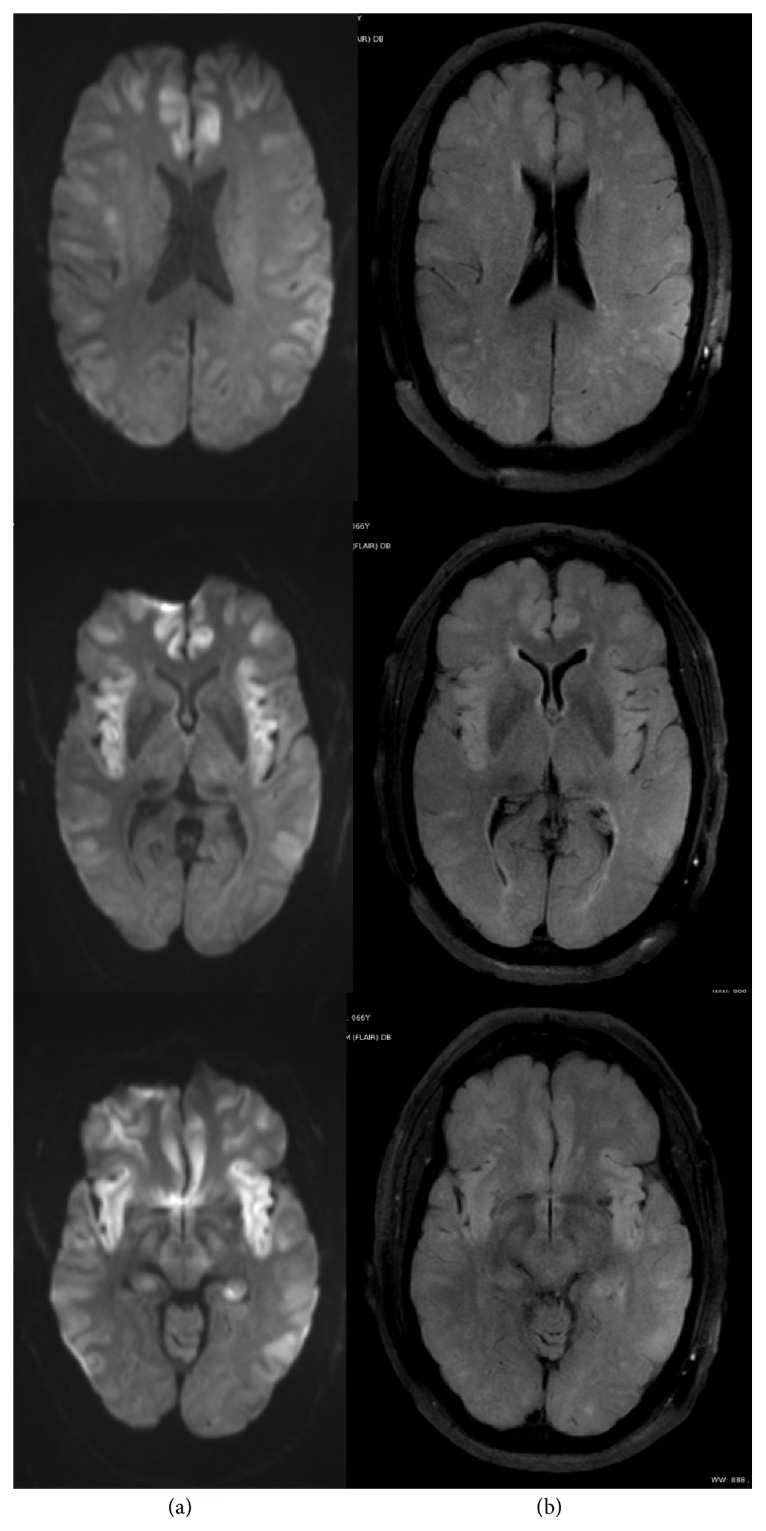
MRI of the brain ((a) DWI sequence, (b) FLAIR sequence) on day 3 after presentation, showing extensive areas of restricted diffusion with associated FLAIR hyperintensity involving bilateral temporal lobes, bilateral insular, bilateral frontal, and parietal regions in cortical and subcortical areas and diffuse mild effacement of the cerebral sulci.

**Figure 2:**
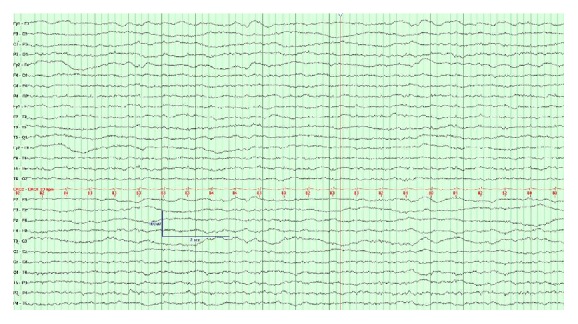
EEG on hospital day 3, showing theta frequency slowing and artifacts from spontaneous horizontal and vertical eye movements but no triphasic activity and no epileptiform activity. HFF 70 Hz, LFF 1 Hz; sensitivity and time base indicated on image.

An MRI of the brain on day of transfer showed extensive areas of restricted diffusion with associated FLAIR hyperintensity involving bilateral temporal lobes and bilateral insular, bilateral frontal, and parietal regions in cortical and subcortical areas and diffuse mild effacement of the cerebral sulci (Figure 1), without enhancing lesions. Continuous electroencephalogram (cEEG) for a duration of 96 hours showed continuous, irregular, generalized low voltage slowing (Figure 2); however, intermittently observed facial and lip twitching did not have an electrographic correlate on cEEG. The MRI findings of symmetric grey matter involvement in the abovementioned areas, and in absence of hypoxic-ischemic insult and seizure activity, were deemed most consistent with hyperammonemic encephalopathy.

The patient underwent continuous venous hemofiltration for two consecutive days followed by regular hemodialysis for two days, with sustained correction of ammonia to levels between 29 and 67 *μ*g/dL after day 3. Liver function panel and coagulation parameters remained within normal limits. Due to poor neurological examination and concern for elevated intracranial pressure (ICP), an ICP monitor was placed, revealing three occurrences of ICP elevation to 28–30 mmHg, which were treated effectively with mannitol boluses. The patient's nutrition was modified to a low protein, high glucose formula to avoid catabolism of endogenous protein. Two days after normalization of ammonia levels, the patient opened his eyes and started to follow commands and move his extremities, and the ICP monitor was discontinued. He was extubated on hospital day 10, at which time he was alert and conversant, without focal motor deficits, yet remained confused.

A computed tomography of the abdomen with venogram did not reveal a portosystemic shunt. An amino acid panel to evaluate inborn errors of metabolism, specifically urea-cycle disorders, was sent (see Table 1). Urine orotic acid excretion was significantly elevated (>900 mmol/mol creatinine; reference range: <2), strongly indicative of a biochemical diagnosis of ornithine transcarbamylase (OTC) deficiency. Genetic testing revealed a pathogenic variant, c.118 C > T, which has been described to cause a mild form of OTC deficiency [3]. In our patient, OTC deficiency was likely unmasked due to a combination of factors: treatment with antibiotics, intake of multiple vitamin supplements, which included red yeast rice, treatment with steroids, and potential interactions of red yeast rice with antibiotics and other herbal supplements.

The patient was maintained on low protein diet and discharged to rehabilitation on hospital day 20 and to home 6 days later, with persistent mild cognitive impairment especially in fluency and memory. A repeat MRI of the brain was obtained 3 weeks after the initial MRI and showed interval decrease in diffusion restriction and FLAIR hyperintensities and resolution of the diffuse mild effacement of the cerebral sulci. He continued to improve clinically and returned to work full time 10 weeks after his initial presentation. At a 3-month follow-up visit, he had returned to his premorbid baseline functional status. At a 6-month follow-up visit, he continued to do well with maintenance of low normal ammonia levels through low protein diet.

## 3. Pathophysiology of Hyperammonemia

### 3.1. Ammonia in the Healthy Human

Main sources of ammonia are colon (through bacterial metabolism of proteins and urea) and small intestine (through bacterial degradation of glutamine) [4]. In a healthy human, the main metabolic route is uptake of ammonia by periportal hepatocytes followed by urea synthesis via the urea cycle [5]. Ammonia that escapes this pathway is converted to glutamine in perivenous hepatocytes [6]. Hepatic transformation of ammonia into urea and subsequent excretion of urea via colon or kidneys prevent entrance of ammonia into the systemic circulation [2]. If the hepatic metabolic capacity is exceeded, or if ammonia bypasses the liver by shunting of blood, circulating ammonia levels increase and elimination of ammonia is shifted to kidneys, brain, and skeletal muscle. Ammonia that reaches the brain can be metabolized by forming glutamine from glutamate [2], mostly in astrocytes with subsequent transfer of glutamine to neurons, and deamination of glutamine resulting in formation of the neurotransmitter glutamate. Muscle adds to the detoxification process by ammonia uptake and synthesis of glutamine [4]. Ammonia excretion through the kidneys, usually accounting for excretion of 30% of ammonia, can be upregulated to 70% [2].

### 3.2. Ammonia in the Brain and Pathophysiology of Hyperammonemic Encephalopathy

Ammonia penetrates the blood-brain barrier through either passive diffusion or mediated transport [7]. While the exact pathogenesis of neurotoxicity is still elusive, ammonia is believed to play a major role by affecting neuronal function as well as creation of brain edema, each of which can contribute to the development of encephalopathy. Ammonia directly affects neuronal electric activity by inhibiting the generation of both excitatory and inhibitory postsynaptic potentials [8]. Increased cerebral uptake of neutral amino acids, resulting from increased amino acid transport in setting of hyperammonemia, can disturb synthesis of the neurotransmitters dopamine, norepinephrine, and serotonin [9]. Enhanced ammonia metabolism in astrocytes leads to an increase in their production of reactive nitrogen or oxygen species [2] and increased intracellular osmolarity, eventually resulting in brain edema [10]. Furthermore, elevated extracellular glutamate levels, a result of ammonia-induced glutamate release and impaired glutamate clearance, may cause overstimulation of N-methyl-D-aspartate (NMDA) receptors. NMDA receptor activation then triggers nitric oxide synthetase which leads to increased synthesis of the vasodilator nitric oxide [11]. Subsequent resultant intracerebral vasodilatation may contribute to the increase in intracranial pressure. Additionally, astrocyte swelling triggers inflammatory cascades, apoptosis, and metabolic pathways that lead to elevated lactate, cerebral edema, and loss of cerebral autoregulation [12].

### 3.3. Noncirrhotic Hyperammonemia

The differential diagnosis of hyperammonemia that is not associated with severe liver disease largely falls into one of two categories: increased ammonia production or decreased ammonia elimination.

### 3.4. Increased Ammonia Production

Increased ammonia production has been observed in hematooncological disorders, organ transplantation, infections, or states of increased catabolism or protein load. The exact mechanism for hyperammonemia in patients with hematooncological disorders is unknown. Myeloma cells have been shown to produce excess ammonia due to increased amino acid metabolism, and plasma cell infiltration of the liver can lead to a portosystemic shunt [13, 14]. In leukemia patients, occurrence of idiopathic hyperammonemia has been described hours to days after initiation of intensive chemotherapy, often followed by progression to coma and death [15]. Similarly, severe hyperammonemia with frequent lethal course has been described in patients with hematological malignancies treated with bone marrow transplantation [16]. Hyperammonemia has also been described in rare cases after heart-lung or lung transplantation [17, 18]. Pathogenesis of hyperammonemia in these situations is believed to be multifactorial involving increased protein catabolism, parenteral nutrition, gastrointestinal hemorrhage, sepsis or mucositis [19], and transient acquired enzyme reductions affecting urea synthesis [20], as well as drug effects from chemotherapy agents [20].

Infections with urea-producing bacteria (*Proteus mirabilis*,* Escherichia coli*,* Klebsiella* species,* Providencia rettgeri*,* Morganella morganii*, and diphtheroids) can lead to noncirrhotic hyperammonemic encephalopathy, mostly reported in children with congenital urinary tract abnormalities and urinary stasis [21–23]. However, cases have been reported in adults with urinary retention and neurogenic bladder, or even in absence of urinary tract abnormalities [24]. Ammonia production in the setting of urinary tract infection alkalinizes the urine with subsequent increase of the fraction of ammonium ions [21]. The ammonium ion is less permeable than neutral ammonia and cannot diffuse back into urine and hence escapes detoxification in the liver by uptake into the systemic circulation via venous drainage from the bladder [23]. Furthermore, systemic mycobacterium or mycoplasma infections in organ recipients as well as herpes simplex virus infection in neonates have been described to lead to hyperammonemia [25–27].

Increased ammonia production also occurs during increased muscle catabolism, such as with seizures, starvation, or trauma [5, 28, 29]. However, symptomatic hyperammonemia usually only occurs in patients with underlying urea-cycle disorders [29]. Similarly, total parenteral nutrition (TPN), which contains a high protein load, has been reported to unmask a long-term asymptomatic urea-cycle disorder [30] or occur with TPN containing only essential amino acids with subsequently impaired ammonia detoxification due to absence of ornithine [31].

### 3.5. Decreased Ammonia Elimination

Inborn errors of metabolism (IEMs) that can cause hyperammonemia include urea-cycle disorders, organic acidurias, carnitine deficiency from defects in fatty acid oxidation, dibasic aminoaciduria, and defects in pyruvate metabolism [6]. While most IEMs present during the neonatal period or early childhood, some, especially urea-cycle disorders, can present in adults. Every enzyme in the urea cycle, carbamoyl phosphate synthetase (CPS), ornithine transcarbamylase, argininosuccinate synthetase (ASS), argininosuccinic acid lyase, and arginase, can be affected by an inherited deficiency [32–34]. OTC deficiency, which is inherited in an X-linked recessive manner, is the most common one, with an estimated prevalence of 1 : 14.000 [35]. Late presentations and phenotypic variance are widely known for OTC deficiency. Mild OTC deficiency can remain largely asymptomatic until an inciting event unmasks the deficiency and leads to symptomatic hyperammonemia [36, 37]. CPS deficiency, N-acetyl glutamine synthetase, and type II citrullinemia also can be present in adulthood [38–40]. Precipitating factors for clinical manifestation of these deficiencies include infections [23, 39], TPN [30], gastrointestinal hemorrhage [41], or valproate intake [42].

Ammonia elimination can be significantly decreased in presence of a portosystemic shunt. Congenital portosystemic shunts are a rare cause of noncirrhotic hyperammonemia, and shunt volume determines time of manifestation, with increased prevalence in patients above the age of 60 [43]. An acquired form of noncirrhotic portosystemic shunt that can lead to hyperammonemic encephalopathy is portal vein thrombosis [44].

Ureterosigmoidostomy is another anatomic situation that can lead to hyperammonemia, due to increased ammonia formation by bacterial degradation after urine excretion directly into the sigmoid colon [45]. While this can occur in liver-healthy individuals, for example, due to coprostasis or infection with urea-splitting bacteria [46], most cases occur in the setting of hepatic failure [47].

### 3.6. Drug-Induced Hyperammonemia

Drug-induced hyperammonemia can result from interference with the urea cycle or enhancement of renal release of ammonia into the systemic circulation. Valproic acid is the most well known [48], but others include carbamazepine [49], sulfadiazine [50], ribavirin [51], salicylates [52], and glycine [53].

The exact pathogenesis of hyperammonemia due to valproic acid is unclear, but it has been suggested that the mechanism is through inhibition of glutamate uptake by astrocytes [48]. The reported prevalence of valproic acid induced hyperammonemia is as high as 35–45% and seems to be higher in patients with carnitine deficiency or with congenital urea-cycle enzymatic defects [54]. Symptoms can present as early as 2 weeks into therapy [6] or as late as several years later [55]. Patients can be asymptomatic with mildly elevated liver enzymes or present with cognitive dysfunction, coma, or severe hepatotoxicity [48, 56]. Serum valproic acid levels can be normal and do not correlate with the level of hyperammonemia or symptoms [56]. Furthermore, ammonia levels do not correlate with the severity of encephalopathy [32].

## 4. Clinical Presentation of Hyperammonemia

Symptoms can range from mild, such as irritability, headache, and vomiting, to severe with encephalopathy, seizures, ataxia, and coma. Depending on the underlying etiology, the symptoms can fluctuate and episodically occur, often precipitated by increased protein intake, drugs, or infections [57]. Psychiatric manifestations, such as manic episodes or psychosis, may be seen in chronic manifestations of late-onset presentations of inborn errors of metabolism [55, 57]. Seizures, cerebral edema, and herniation are manifestations of acute hyperammonemia and usually occur with ammonia levels exceeding 200 *μ*mol/L [58]. The difference between acute and chronic hyperammonemia is believed to lie in the effect of glutamine on the brain [5]. In patients with acute liver failure, a strong association of arterial ammonia levels higher than 200 *μ*g/dL with cerebral herniation has been shown [59].

## 5. Diagnosis

### 5.1. Laboratory Workup

To accurately determine the serum ammonia level, the blood sample must be obtained and handled correctly. Hemolysis, inappropriate handling and transportation of the specimen (the specimen needs to be placed on ice), and delayed analysis all can falsely elevate ammonia levels. Both venous and arterial blood can be sampled for measurement of ammonia [60]. In patients with hepatic failure who are suspected to have hepatic encephalopathy, measurement of ammonia remains controversial. While there is a correlation between ammonia levels and severity of hepatic encephalopathy, absolute elevation of ammonia is inconsistent [60]. Furthermore, hepatic encephalopathy is only directly related to arterial ammonia levels up to about twofold increase above normal; beyond that, the grade of hepatic encephalopathy is dependent on the partial pressure of gaseous ammonia rather than total serum ammonia [61]. However, this may not necessarily be applicable to hyperammonemia in absence of hepatic failure.

A value of more than 100 *μ*mol/L (175 *μ*g/dL) in older children and adults should trigger further investigation, especially in setting of normal liver function [62]. For isolated hyperammonemia, first evaluations include acid-base status, serum bicarbonate, sodium, chloride, and urine ketones [63]. Acidosis raises the possibility of organic acidemias whereas respiratory alkalosis may be an indication of a urea-cycle disorder. In absence of acidosis, the concentrations of amino acids in blood and urine should be evaluated [62]. A nondiagnostic amino acid profile would trigger evaluation for urine orotic acid, as orotic aciduria is found in patients with OTC deficiency. If both amino acid profile and orotic acid testing are nonrevealing, the diagnosis likely is CPS deficiency or, much more rarely, NAGS deficiency [62]. In patients with episodic symptoms, the diagnosis may be elusive until reevaluation during crisis [63]. Ultrasonography, abdominal computed tomography, and magnetic resonance imaging can all demonstrate portohepatic shunts [64]. A liver biopsy should be considered to measure enzyme levels in hepatocytes if an inborn error of metabolism is suspected [32]. Liver biopsy can furthermore be invaluable in excluding cirrhosis of any cause or diagnose other hepatic diseases.

### 5.2. Neuroimaging

Magnetic resonance imaging (MRI) has provided insight into characteristic patterns observed in hyperammonemic encephalopathy. Diffusion restriction is found predominantly cortical and often diffuse and symmetric, with corresponding areas of FLAIR hyperintensities [65]. Insular cortex and cingulate gyrus are most commonly affected, but it remains unclear why these areas are particularly susceptible to hyperammonemia [66, 67]. Thalamus, parietal, frontal, temporal, or occipital cortices involvement can be seen in variable extent and may be asymmetric [66, 67]. There is one case report of thalami and basal ganglia involvement, and involvement of the occipital cortices is considered rare [65]. In neonatal cases of inborn errors of metabolism with resultant hyperammonemia, a slightly different pattern was described with involvement of the lentiform nuclei, insular sulci, and perirolandic regions possibly due to those areas being more metabolically active in the neonatal period [68]. Radiographic abnormalities may be detected even if ammonia is only slightly elevated [65, 66]. The extent of the imaging findings may depend on severity and duration of hyperammonemia and predisposing susceptibility to the metabolic insult [68]. Cortical changes in hyperammonemic encephalopathy have been shown to be potentially reversible [69] but can also result in variable amounts of atrophy in the cingulate and insular cortex [66, 68].

Differential diagnosis includes posterior reversible encephalopathy syndrome, seizure activity, metabolic and hepatic encephalopathy, and diffuse hypoxic-ischemic injury [66]. Exclusion of an overlapping effect of hypoxic injury or seizure activity on imaging findings may be difficult, but very symmetric involvement of cingulate and insular gyri favor a toxic encephalopathy [66].

## 6. Treatment

Acute treatments of hyperammonemia are geared towards lowering the blood ammonia level and manage seizures, cerebral edema, and elevated intracranial pressure. Continuing treatments may target the specific etiology of hyperammonemia such as inborn errors of metabolism and vary with the condition.

### 6.1. Ammonia-Lowering Treatment

Nonabsorbable disaccharides, which decrease intestinal ammonia production and absorption, are an established first-line therapy for hepatic encephalopathy and the mainstay of treatment for chronic encephalopathy; however, they do not affect mortality [70]. Antibiotics that decrease enteric ammonia production by reducing the amount of urease-producing bacteria are another treatment option. Neomycin, an antibiotic and glutaminase inhibitor, has been FDA approved for use in acute hepatic encephalopathy but is also commonly used with lactulose as off-label treatment for chronic encephalopathy [71]. Rifaximin, a nonabsorbable antibiotic derivative, is used as first-line or in addition to nonabsorbable disaccharides in acute or chronic encephalopathy [72]. Sodium benzoate or sodium phenyl acetate enhances alternative pathways of ammonia metabolism and subsequent excretion via urine [33]. Emergent hemodialysis can be used to rapidly, though temporarily, decrease serum ammonia, with conventional hemodialysis offering the highest ammonium clearance rate of all dialysis methods [73].

### 6.2. General Management

Data and practices of management for elevated ICP and cerebral edema are largely available from observations in (fulminant) hepatic failure and may not apply to isolated hyperammonemia. Usual management includes hyperosmolar agents and propofol sedation [74]. While mannitol used to be the mainstay of therapy despite doubts about efficacy [75], hypertonic saline has gained more attention [76]. For medically refractory intracranial hypertension, decompressive craniectomy can be considered for a potential positive outcome [77]. ICP monitoring and transcranial Doppler sonography may be used to assist in diagnosis and monitoring of treatment effect [78], but ICP monitoring has been shown to have potential detrimental effects in absence of mortality benefit [79].

### 6.3. Specific and Ongoing Treatments

Introduction of a diet with a favorable calorie-to-nitrogen ratio and restriction of exogenous protein is a supporting measure [80]. L-Carnitine plays a critical role in the intermediary metabolism of fatty acids and their transport across mitochondrial membranes and has been shown to be of use in treatment of inborn errors of metabolism [81]. Supplementation with L-carnitine in urea-cycle disorders may lower the frequency of attacks [82]. While primary treatment for valproic acid induced hyperammonemia is withdrawal of the drug and limitation of potentiating drugs such as phenobarbital and phenytoin [83], treatment of valproic acid induced hyperammonemia with L-carnitine has been reported to improve both symptoms and survival [84].

L-Ornithine-L-aspartate increases muscle ammonia metabolism and has been shown to be beneficial in clinically manifest hepatic encephalopathy [85]. Arginine, the immediate precursor of ornithine, can serve as treatment of hyperammonemia in urea-cycle disorders by replenishing the urea-cycle substrates [86]. Liver transplantation is an important treatment modality in urea-cycle disorders, with high survival rates that are superior to survival in liver transplantation for other diseases [87]. Portosystemic shunts may be obliterated surgically or by interventional radiological techniques but, depending on the type, may also require liver transplantation [88].

## 7. Discussion

Several case reports have described acute onset of hyperammonemia due to late-onset inborn errors of metabolism in previously healthy adolescents or adults. The majority of these cases are fatal [89–92]. Panlaqui et al. report a case of a 48-year-old man with preceding subacute cognitive decline and sudden encephalopathy, an ammonia level of 390 *μ*mol/L, MRI findings of diffuse cortical edema and T2 hyperintensities, successfully treated with hemodialysis, and however persistent significant cognitive deficits at 6-month follow-up [93]. Mahmood and Nugent describe a 35-year-old woman with late-onset OTC deficiency unmasked by gastrointestinal hemorrhage, presenting with encephalopathy [94]. A CT of the head in this patient was reportedly normal, and ammonia level was 593 *μ*g/dL [94]. Wendell et al. report successful management of ICP in a 37-year-old female with known OTC deficiency, ammonia level of 904 *μ*mol/L, and progressive cerebral edema on head CT eventually requiring decompressive craniectomy [77], with good outcome. U-King-Im et al. discuss a series of four patients with acute adult hyperammonemic encephalopathy who presented with seizures and decreased level of consciousness and plasma ammonia levels between 55 and 168 *μ*mol/L [66]. Unlike our patient, these four described patients all had severe systemic disease or hepatic failure, namely, (1) status after heart-lung transplantation with multiple organ dysfunction and sepsis, (2) fulminant acute hepatic failure, (3) severe sepsis in setting of chronic cirrhosis, and (4) severe sepsis in a hepatic transplant patient with hepatorenal syndrome. All four patients had MRI findings delineating the characteristic findings of hyperammonemia. Only one patient had a good outcome [66]. Rosario et al. describe three patients with cirrhosis, alcoholic hepatitis, and acetaminophen hepatotoxicity [67], with characteristic MRI findings. Only the patient with acetaminophen toxicity, whose ammonia level had been 217 *μ*mol/L, had a good outcome [67].

We therefore present a novel and unique case of an adult with late-onset OTC deficiency, with very severe clinical manifestation and very high ammonia levels, as well as extensive MRI findings characteristic for hyperammonemic encephalopathy. Absence of status epilepticus and only mild transient ICP elevation point to hyperammonemia as the main factor in causing this patient's encephalopathy and imaging findings. Our case highlights that extensive imaging findings can be reversible in late-onset OTC deficiency and that outcome can be excellent if correctly diagnosed and aggressively and rapidly treated.

## 8. Conclusion

Hyperammonemia should be considered in the differential diagnosis for encephalopathy and seizures, especially in presence of MRI findings of bilateral symmetric involvement of insular and cingulate cortices. In absence of hepatic dysfunction, hyperammonemia can be caused by increased ammonia production or decreased ammonia excretion. Aggressive management with ammonia-lowering measures, cerebral edema, and increased intracranial pressure is warranted.

## Figures and Tables

**Table 1:** Results of serum amino acid testing.

Amino acid	Result (nmol/mL)	Reference range (nmol/mL)
Phosphoserine	0	<18
Phosphoethanolamine	<2	<12
*Taurine*	*24 (L)*	42–156
Asparagine	40	37–92
*Serine*	*47 (L)*	63–187
*Hydroxyproline*	*2 (L)*	4–29
*Glycine*	*93 (L)*	126–490
Glutamine	892	371–957
Aspartic acid	2	<7
Ethanolamine	<7	<67
Histidine	61	39–123
*Threonine*	*43 (L)*	85–231
Citrulline	22	17–46
Sarcosine	1	<5
Beta-alanine	13	<29
*Alanine*	*88 (L)*	200–579
Glutamic acid	43	13–113
1-Methylhistidine	0	<28
3-Methylhistidine	3	2–9
Argininosuccinic acid	0	<2
Carnosine	0	<1
Anserine	0	<1
Homocitrulline	1	<2
*Arginine*	*28 (L)*	32–120
Alpha-aminoadipic acid	2	<3
Gamma-amino-n-butyric acid	0	<2
Beta-aminoisobutyric acid	1	<5
*Alpha-amino-n-butyric acid*	*77 (H)*	9–37
Hydroxylysine	0	<2
*Proline*	*81 (L)*	97–368
*Ornithine*	*13 (L)*	38–130
Cystathionine	<1	<5
Cystine	36	3–95
*Lysine*	*283 (H)*	103–255
Methionine	41	4–44
Valine	179	136–309
Tyrosine	40	31–90
Isoleucine	54	36–107
Leucine	93	68–183
Phenylalanine	48	35–80
Tryptophan	35	29–77
Allo-isoleucine	0	<5
